# Microbiota Modulating Nutritional Approaches to Countering the Effects of Viral Respiratory Infections Including SARS-CoV-2 through Promoting Metabolic and Immune Fitness with Probiotics and Plant Bioactives

**DOI:** 10.3390/microorganisms8060921

**Published:** 2020-06-18

**Authors:** Tanvi Shinde, Philip M Hansbro, Sukhwinder Singh Sohal, Peter Dingle, Rajaraman Eri, Roger Stanley

**Affiliations:** 1 Centre for Food Innovation, Tasmanian Institute of Agriculture, University of Tasmania, Launceston, TAS 7250, Australia; 2 Gut Health Research Group, School of Health Sciences, College of Health and Medicine, University of Tasmania, Launceston, TAS 7250, Australia; rajaraman.eri@utas.edu.au; 3 Centre for Inflammation, Centenary Institute, Sydney, NSW 2050, and University of Technology Sydney, Faculty of Science, Ultimo, NSW 2007, Australia; Philip.Hansbro@uts.edu.au; 4 Respiratory Translational Research Group, Department of Laboratory Medicine, School of Health Sciences, College of Health and Medicine, University of Tasmania, Launceston, TAS 7248, Australia; sukhwinder.sohal@utas.edu.au; 5 Dingle Wellness, South Fremantle, WA 6162, Australia; peter@drdingle.com

**Keywords:** COVID-19, dietary fiber, innate immune response, probiotic, synbiotic, functional foods, short-chain fatty acids (SCFAs), dysbiosis, gut microbiota

## Abstract

Viral respiratory infections (VRIs) can spread quickly and cause enormous morbidity and mortality worldwide. These events pose serious threats to public health due to time lags in developing vaccines to activate the acquired immune system. The high variability of people’s symptomatic responses to viral infections, as illustrated in the current COVID-19 pandemic, indicates the potential to moderate the severity of morbidity from VRIs. Growing evidence supports roles for probiotic bacteria (PB) and prebiotic dietary fiber (DF) and other plant nutritional bioactives in modulating immune functions. While human studies help to understand the epidemiology and immunopathology of VRIs, the chaotic nature of viral transmissions makes it difficult to undertake mechanistic study where the pre-conditioning of the metabolic and immune system could be beneficial. However, recent experimental studies have significantly enhanced our understanding of how PB and DF, along with plant bioactives, can significantly modulate innate and acquired immunity responses to VRIs. Synbiotic combinations of PB and DF potentiate increased benefits primarily through augmenting the production of short-chain fatty acids (SCFAs) such as butyrate. These and specific plant polyphenolics help to regulate immune responses to both restrain VRIs and temper the neutrophil response that can lead to acute respiratory distress syndrome (ARDS). This review highlights the current understanding of the potential impact of targeted nutritional strategies in setting a balanced immune tone for viral clearance and reinforcing homeostasis. This knowledge may guide the development of public health tactics and the application of functional foods with PB and DF components as a nutritional approach to support countering VRI morbidity.

## 1. Introduction

Viral respiratory infections (VRIs) are a major public health issue. Annually, they induce infectious diseases that result in enormous severe morbidity and mortality globally. Major viral pathogens include the influenza virus, respiratory syncytial virus (RSV), coronavirus, adenovirus, and rhinovirus [1]. The influenza virus is the major cause of seasonal respiratory infections, causing annual epidemics with an estimated 3 to 5 million severe illnesses and 290,000 to 600,000 deaths worldwide [2]. However, other viruses that are associated with lower mortality also cause a huge economic burden owing to their high morbidity [1]. The constant threat of the emergence of novel subtypes and strains of viruses creates an even greater risk to society. This has been clearly demonstrated by the recent pandemic, with the novel coronavirus (CoV)-2 causing severe acute respiratory syndrome (SARS-CoV-2) in susceptible people. First reported in late December 2019 in Wuhan, China, and spreading rapidly across the globe, >3.5 million cases have been confirmed and >250,000 deaths have been reported [3]. The human-to-human transmission of the novel virus, the induction of COVID-19 pneumonia and acute respiratory distress syndrome (ARDS) [4], and its continual geographic expansion serves as an important reminder of our vulnerability to emerging viral infections.

Vaccination to engage the acquired immune system is considered the most effective available means of protecting populations against viral diseases in general. However, rapid antigenic changes in viruses, leading to the emergence of novel strains, hampers efforts to provide adequate strain-specific timely protection against viral diseases. Moreover, the low to moderate effectiveness of vaccines in at-risk populations—for instance, against influenza [5,6]—accounts for its high annual mortality. Immunocompromised individuals show a higher propensity towards acquiring severe respiratory infections and mortality [7,8]. Sub-optimal immune health can be related to age [9], chronic inflammatory conditions [10], metabolic dysfunction [11], or stress [12]. The decline in function compromises the ability of immune cells to counter infections and can lead to the dysregulation of the immune response. Most patients with SARS-CoV-2 infections have mild to moderate illness accompanied by shortness of breath after one week [13]. In severely ill patients, the infection progresses rapidly to acute respiratory failure, ARDS, metabolic acidosis, and septic shock [13]. The shortage of key equipment, including ventilators needed to care for critically ill patients during the COVID-19 pandemic [14], has also led to the need for the rationing of medical equipment and interventions [15]. This underscores the urgent need for directing research efforts towards effective approaches for blunting acute responses to infections particularly in at-risk populations.

There is an increasing consensus on the roles of commensal microbiota in shaping host immunity [16,17,18,19,20,21]. This highlights the opportunity for the modulation of microbiota and its functions to regulate host immune responses against viral infections. Considerable evidence supports the potential of functional food components, such as probiotic bacteria (PB) and prebiotic dietary fiber (DF), in inducing beneficial changes in microbial composition and metabolic function. Thus, the application of PB, DF, and other plant bioactives to confer metabolic and immune benefits could be a pragmatic prophylactic nutritional strategy capable of being widely implemented. This review aims to identify evidence relating to potential mechanisms that could be deployed as preventive strategies and adjuvants to impart immune fitness against the immunopathology of VRIs.

## 2. Immunopathogenesis of VRIs

**Figure 1:**
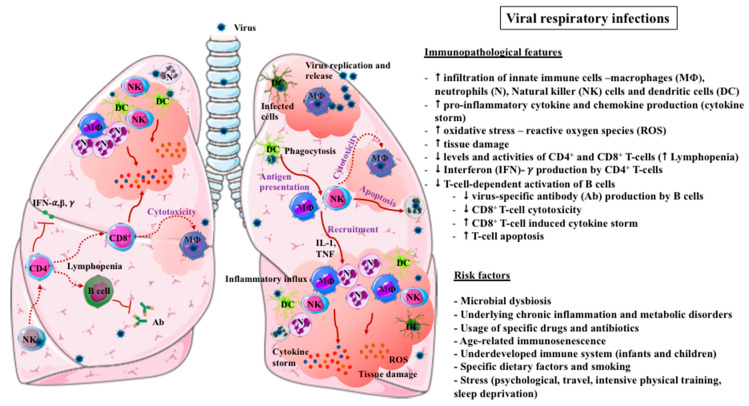
Immunopathological features and risk factors associated with viral respiratory infections.

Understanding the immunopathological features of VRIs is crucial to facilitating the development of effective strategies to confer protection and designing specific treatments targeted at improving immune functions in response to VRIs and associated damage (Figure 1). Evidence from influenza virus infections and three major beta coronavirus (CoV) pandemic infections—Middle East Respiratory Syndrome (MERS), Severe Acute Respiratory Syndrome (SARS)-CoV-1, and COVID-19—underscores some key immunopathological features of the diseases [22,23]. These include the dysregulation of acute inflammatory responses involving macrophages, neutrophils, dendritic cells (DCs), toll-like receptors (TLRs), cytokines, chemokines, and CD4+ and CD8+ T-cells, as well as tissue remodeling processes and the role of bacterial superinfection [22]. Infection with the influenza virus induces heightened immunological responses that are necessary for viral clearance, including the influx of innate immune cells and the overproduction of cytokines and chemokines. While triggering cytotoxic mechanisms to destroy viral-infected cells is also necessary, this can be detrimental, leading to pulmonary immunopathology [22,24,25]. Tissue damage induced by uncontrolled innate immune responses and excessive neutrophil infiltration upon influenza virus infection has been linked to severe morbidity and mortality [22]. Elevated myeloperoxidase (MPO) activity (congruent with increased neutrophil influx) is confirmed to cause tissue injury and endothelial damage in the lungs [26]. The development of fibrosis in SARS-CoV-2-infected lungs in COVID-19 pneumonia has also been reported [27]. Marked neutrophil infiltration in the lungs of COVID-19 patients has been implicated in inducing a “cytokine storm” [28,29]. The neutrophil-to-lymphocyte ratio (NLR) has been reported to predict severe illness in patients with COVID-19 in the early stages of infection [13]. This agrees with another study that confirmed a strong association between elevated NLR with increased abdominal obesity-induced systemic inflammation in the older Spanish population [30]. Thus, NLR may be used as a prognostic factor for disease severity in VRIs that could facilitate an early detection of severity and the rapid implementation of interventions to aid patient responses to infection.

Virus-induced oxidative stress via the generation of reactive oxygen species (ROS) is also a factor that increases the expression of proinflammatory cytokines and chemokines [31] and sensitizes lung cells to bacteria toxin-mediated necroptosis [32]. The heightened neutrophil influx and cytokine storm coupled with subsequent ARDS correlates with multi-organ damage and mortality among COVID-19 patients [28]. The markedly increased serum concentrations of interleukin (IL)-2R, IL-6, tumor necrosis factor (TNF)-α, and IL-10 that occur more in patients with severe COVID-19 than in moderate cases also suggests an association between the cytokine storm and the disease severity [29]. The dysregulated responses of other immune cells, including macrophages and DCs, are also associated with the pulmonary immunopathology of influenza [22]. These observations indicate that the over-exuberant activation of innate immunity can be detrimental, and strategies to regulate their optimal functioning should be targeted.

Adaptive immune components and CD4+ and CD8+ T-cells are essential for virus clearance and immune regulation. SARS-Co-V2 infection has been reported to cause significant alterations in circulating lymphocytes and T-cell subsets [29]. Lymphopenia, particularly in CD4+ and CD8+ T-cells, as well as reduced interferon (IFN)-γ production by CD4+ T-cells but not CD8+ T cells or natural killer cells are associated with the increased severity of COVID-19 [29]. CD4+ T-cells regulate the immune response by orchestrating the deletion and amplification of immune cells, especially CD8+ T-cells. CD4+ T-cells mediate virus-specific antibody production via the T-cell dependent activation of B cells [33]. For influenza infections, it was shown that the production of IFN-γ by CD4+ T-cells was required for viral clearance, thus ameliorating the immunopathology [22]. In contrast, CD8+ T-cells have cytotoxic effects through cytolytic activities against the target cells or the secretion of cytokines and chemokines [34]. MERS-CoV infects T-cells from peripheral blood and human lymphoid organs and induces their apoptosis, thus contributing to the lymphopenia [35], which is also observed for SARS-CoV-1 [36]. Hence, strategies that promote tight immune regulation and effectively mount adequate levels of innate and adaptive immune responses for viral clearance and the restoration of immune homeostasis should be prioritized. In addition, considering sub-optimal immune activity in immunocompromised patients, developing treatments and prophylactic approaches that promote sufficient immune responses for viral clearance without causing additional damage should be pursued.

## 3. Sub-Optimal Immunity Driven by Microbial Dysbiosis

A well-functioning immune system determines the host immune fitness, which dictates protection against invading pathogens. The immune surveillance system continually monitors for signs of invasion and signals for appropriate immune responses to mount a defense against specific pathogens. Appropriate immune responses maintain strength and/or return to immune homeostasis after an external challenge [37]. Reponses to gut microbiota are known to influence homoeostasis at distant mucosal sites, including the respiratory tract, through a common mucosal immune system [16,38,39,40]. The immunological health of the gut, primarily mediated by the microbiota, influences lung health via the “gut-lung axis” [39,40,41,42,43]. In addition, microbial communities inhabiting the mucosal surfaces of the respiratory tract also contribute towards host defense against VRIs [17,18]. Acute VRIs are associated with microbial dysbiosis in these communities, thus affecting the optimal functioning of the immune system. Alterations in the microbiota during influenza virus infection contributes to the pathogenesis of secondary bacterial infections, thus increasing the severity of the clinical course in the absence of appropriate immune responses [44].

Additionally, alterations in immune functions associated with chronic inflammation and related metabolic dysfunctions are also known to drive impairment in innate and acquired immune functions in the host [10,11,45]. The failure to effectively mount appropriate immune responses against pathogens renders the host susceptible to infection. The prevalence of comorbidities (including chronic pulmonary diseases, diabetes, hypertension, and cardiovascular diseases) and old age predispose one to infection, the development of ARDS and pneumonia, and a high mortality in COVID-19 patients [46]. Similar factors have been noted for previous infections, such as with influenza virus [7,8,47]. Chronic inflammation and antibiotic use are known to accompany disturbances in the gut microbiota, resulting in dysbiosis and thus aggravating immune dysfunctions [17,48]. Moreover, ageing-related changes in the composition and function of gut microbiota are associated with immunosenescence, a state of gradual deterioration of the immune system [9]. Immunosenescence affects both the innate and adaptive arms of the immune system. This results in a progressive reduction in the ability to mount effective cellular and antibody responses against infections and vaccination in the elderly, increasing their vulnerability to infectious agents [49]. Children and infants are more prone to VRIs due to their immature immune systems. The early-life microbial colonization of the gut and airways influences their susceptibility to severe VRIs [50]. For adults, lifestyle factors including cigarette smoking [51,52,53] and the consumption of a Westernized diet, characterized by low dietary fiber content [54,55] and high fat intake [56], have been associated with promoting the exacerbations of viral infections due to impairment in immune functions. Smoking upregulates the angiotensin-converting enzyme-2 (ACE2) receptor, which has been reported to be the receptor for SARS-CoV-1 and SARS-CoV-2 [53,57]. Smoking is established as the primary etiological factor for chronic obstructive pulmonary disease (COPD) and the patients with COPD are significantly vulnerable to the increased frequency and progression of respiratory viral-induced exacerbations and mortality [24]. In addition, stress-related dysbiosis also has a role in rendering the host susceptible to VRIs. Psychological, intensive physical training, sleep deprivation, and travel-related stressors can cause gut dysbiosis and are associated with respiratory infections among travelers, military troops, astronauts, and elite athletes [12,58,59,60]. Thus, perturbations in gut and respiratory microbial communities are a major contributor to the deterioration of immune homeostasis and resilience that is critical for protection against VRIs.

## 4. Functional Foods for Immune Fitness

Given the strong links between the impairment of immune functions and severity of viral infections, the development of strategies to support optimal immune function may be effective in protection and prevention of severe morbidity. Increasing evidence exists that supports the roles of gut microbiota and diet in shaping immunity [16,17,18,19,20,21,61,62]. Modulating the composition and metabolic capacity of the microbiome by specific dietary components is a promising strategy to influence immune responses against VRIs. Functional food components including PB, prebiotic DFs and other plant-based bioactive component have been associated with immune benefits, primarily via microbiota modulation and impacting oxidative stress [63,64]. Despite the evidence in promoting health benefits, functional foods have received less attention relative to pharmaceutical agents for application to human VRIs in clinical settings [65]. While functional foods are largely prophylactic and not treatment options for acute medical conditions, there is also uncertainty and even skepticism about their efficacy. This could be principally attributed to variations in the study outcomes of functional food bio-actives. However, while variation in study outcomes is expected from the wide range of functional food components and different intervention study designs, it does not preclude the efficacy of application in specific cases. The experimental evidence elucidating the immune mechanistic efficacy of particular bioactive components and/or combinations has huge potential in guiding public health strategies to develop effective dietary approaches to prevent and/or treat VRIs via promoting immune fitness.

## 5. Probiotics

Probiotics are “live microorganisms that when administered in appropriate doses, confer a benefit to the health of the host” [66]. Many probiotic bacteria are members of the gut microbiota, and some are being increasingly incorporated into foods to improve gut health and wellbeing. Recently, the immunogenic potential of even non-viable probiotic cells, bacterial exopolysaccharides, and spores has also been noted [67,68,69,70,71,72]. Additionally, the ingestion of probiotic-rich food or supplements has been shown to influence immune functions, partly by driving changes in the metabolic activities of endogenous microbiota [73].

The ability of PBs to induce immunomodulation could be mediated either directly through interaction with immune cells or indirectly by supporting the challenged commensal microbiota [74]. Ingested PBs stimulate the immune system and initiate a network of signals mediated by the whole bacteria or their cell wall components. Once administered, they interact with intestinal epithelial cells (IECs) or immune cells associated with the lamina propria through TLRs or other microbial pattern recognition receptors (PRRs) and trigger the production of an array of cytokines and chemokines. These molecules then interact with other immune cells through a complex network of signaling pathways, leading to the activation of the mucosal immune systems. Specific PBs have been demonstrated to enhance T-helper 1 (Th1) and regulatory T-cell (Treg) function [75] and strengthen the epithelial barrier function by increasing mucin production, tight junction proteins, and goblet cells [76,77,78]. In contrast to some earlier reports [79,80,81], current studies support the potency of specific PBs in inducing changes in gut bacterial diversity [82,83,84]. Probiotic-driven benefits are also linked with their capacity to cross-feed other beneficial members of the gut microbiota by digestive activities that release nutrients. They can therefore promote metabolic shifts, including the increased production of SCFAs [77,78,85]. This can have substantial health benefits, as microbiota-derived SCFAs are known to play a significant role in maintaining gut physiology and influencing metabolic balance [86,87]. Thus, PB application offers potential as a strategy for supporting a healthy immune system.

Experimental studies indicate that there can be a substantial potency of specific PB strains in protecting against VRIs. The effects of PBs on the mucosal system are not limited to the intestinal tract, with cross-modulatory effects confirmed in other locations, including the upper respiratory tract. Protection is mediated by the induction of systemic and/or local cellular immune responses [88]. Specific PBs are also known to stimulate the humoral immune response by increasing the numbers of IgA-secreting cells that migrate from Peyer’s Patches to distant mucosal sites such as respiratory glands [89]. Mice models have been instrumental in demonstrating the potential of mechanisms through which probiotics could confer benefits. The oral administration of live or heat-killed probiotic strains of Lactobacillus [90,91,92,93,94,95] and Bifidobacterium [96] in mice has been demonstrated to improve cytokine production against viruses in the lungs or serum. In addition, many studies support the potency of intranasally administered PBs to protect mice against VRIs by stimulating local innate immune responses directly in the respiratory epithelium [92,97,98,99,100,101]. Some studies have also confirmed the excellent efficacy of certain heat-killed strains of Lactobacillus and Bifidobacterium to stimulate the synthesis of virus-specific immunoglobulins and cellular immune responses in respiratory secretions and serum in mice [67,68,69,70]. Similarly, the oral administration of mice with Bifidobacterium breve YIT4064 increased anti-influenza virus IgG antibodies in serum and protected against infection [102]. The sublingual administration of *L. rhamnosus* in influenza-infected mice enhanced mucosal secretory IgA production, T and NK cell activities, and IL-12 levels in the lungs [103], thus supporting local cellular and humoral immune functions. However, while the mice models are indicative, the differences in the evolved biology of humans compared to mice requires caution in translation of the results to impacts on human disease [104]. Direct human trials will be needed to validate the probiotic effects.

Most evidence of PB-related benefits has been demonstrated in the treatment or management of specific pathologies [105,106,107,108]. Studies evaluating the immunomodulatory efficacy and potential prophylactic activity of PBs in non-diseased subjects are minimal. PB supplementation with influenza vaccination improved the vaccine efficacy in healthy adults [109,110], supporting the role of PBs in VRIs. However, the systematic reviews of randomized controlled trials of probiotic supplementation in healthy subjects are not conclusive. Studies and meta-analysis of trials report mixed results [111,112,113]. Differences in the duration of intervention, age of subjects (children, adults, elderly), doses (106–1010 cfu), or matrices (milk, yoghurt, capsule) may partly account for the conflicting results [88]. Differences in susceptibility, or other unaccounted risk factors in otherwise healthy populations, may also explain variations in PB efficacy on influencing health outcomes found in the trials. While changes in immune responses induced by interventions in healthy subjects with generally well-functioning immune systems are difficult to demonstrate, the elderly can have sub-optimal immunity. Infectious diseases are common in elderly populations due to the age-related decline in immune efficacy referred to as immunosenescence [9]. Furthermore, ageing-related microbial dysbiosis is also known to fuel inflammation in the gut, affecting immune regulation abilities [49]. Daily supplementation with *B. lactis* HN019 was reported to enhance NK cell tumoricidal activity and polymorphonuclear (PMN) phagocytic capacity, reinforcing immune resilience against viral infections in healthy elderly subjects. In addition to the decline in the functions and proportions of T and B cells [9], age-related changes have also been reported for innate immune components, including PMN and NK cells [109]. Neutrophils that account for 90–95% of PMN cells in the blood have been confirmed to have reduced chemotaxis and phagocytic activity in the elderly [110]. Despite the increase in the numbers of NK cells with age, their signaling capacity, cytokine production, and up-regulation of co-stimulatory molecules is reported to deteriorate [114]. NK cell immunosenescence has been linked to the higher incidence of viral infections in the elderly [115,116].

In addition to directly influencing immune cell functions, certain PBs may also induce beneficial modulations in gut microbiota that in turn have an impact on immune status. *B. longum* strains have been demonstrated to cause changes in the level of specific *Bifidobacterium* species in the elderly that correlated with TNF-α, TGF-β, and IL-10 levels in serum [117]. The daily consumption of *Bacillus coagulans* BC30 PB spores has been shown to significantly increase the populations of *Faecalibacterium prausnitzii*, with concomitant increases in anti-inflammatory IL-10 levels in elderly subjects [118], supporting the direct correlation confirmed by another study [119]. *B. coagulans* BC30 has been confirmed in ex vivo experiments to increase the T-cell production of TNF-α in response to specific adenovirus and influenza virus exposures [71]. *L. casei* [120] and *L. plantaurm* [121] have also been evaluated and shown to improve influenza virus vaccination in the elderly

Age-related microbial dysbiosis also affects the metabolic capacity of gut microbiota in producing beneficial metabolites, including SCFAs [49,109]. Declines in the populations of *Bacteroides* group, *Bifidobacterium*, *Faecalibacterium*, *Akkermansia*, or *Clostridium cluster* XIVa in the elderly gut is commonly reported [49]. The supplementation of aged mice with *L. acidophilus* DDS-1 resulted in increased relative abundances of beneficial *Akkermansia* and *Lactobacillus* species and enhanced butyrate levels while concomitantly downregulating pro-inflammatory cytokines [82]. Taken together, these studies show that PB application could encourage the restoration of immune functions in the elderly, however more research on the strain-specific effects of PBs is required.

PB-induced benefits in respiratory infections have also been shown in infants and children. Some studies have found specific Lactobacillus and Bifidobacterium PB strains to be beneficial in reducing the number of participants experiencing respiratory infection episodes, their mean duration, antibiotic use, and school or childcare absences [122,123,124]. However, some reported no clear benefit [125,126]. Childcare exposure has been associated with the increased risk of upper respiratory infections along with the immature immune system of children [126]. A meta-analysis confirmed that infants and children who received PBs to prevent common acute illness had a reduced risk of being prescribed antibiotics [124]. A recent systematic review on the benefits of PB-supplemented infant formula concluded that some beneficial effects are possible; however, a lack of existing robust evidence to recommend their routine use was reported [127]. The small amount of data on specific PB strains and outcomes, rather than a genuine lack of effects, was acknowledged. In infants, a PB-supplemented formula (*B. infantis* R0033, *B. bifidum* R0071, and *L. helveticus* R0052) was confirmed to sustain the development of mucosal immunity through effects on secretory IgA (sIgA) production [128]. sIgA accounts for 90% of the immunoglobulins in breast milk and protects the intestinal mucosa and prevents infections mainly by blocking the contact of pathogens with epithelial layer and trapping them within mucus layers [129]. sIgA in the intestine maintains controlled microbial colonization while preserving mucosal homeostasis in newborns [128]. In this context, PBs may benefit the immature immune system of formula-fed newborns that lack the vital first line of defense against pathogens.

## 6. Prebiotic DF

The lower consumption of fruits and vegetables among adults in Western countries is congruent with the epidemic rise in chronic conditions, including respiratory conditions, obesity, diabetes, cardiovascular diseases, and even cancer [130,131]. A “Westernized diet” is strongly associated with chronic local and systemic inflammation, leading to microbial dysbiosis, altered immunity, and weakened gut barrier functions [63,132]. This diet is generally high in refined carbohydrates and fat, with a low DF content from fruits and vegetables. The underlying chronic conditions were noted to account for high risk of morbidity and mortality for the recent MERS, SARS, and COVID-19 major beta coronavirus pandemics [133,134]. Conversely, diets rich in fruits and vegetables have been shown to lower the risk of cardiovascular diseases, metabolic disorders, and gastrointestinal disorders [135,136,137]. Thus, plant-based diets, functional foods, and supplements present a promising strategy for protecting against respiratory infections.

Prebiotic DF from fruits and vegetables is well-established to modulate the gut microbiota, and numerous benefits have been reported in chronic inflammatory and metabolic conditions [138]. Moreover, increased DF consumption is linked to reduced mortality rates in respiratory-related diseases [139] and improved lung function [140]. A few studies report the ability of prebiotic supplements to confer immune response benefits against viral vaccinations. A prebiotic nutritional formula supplemented with fructo-oligosaccharides (FOS), triglycerols, vitamins, and minerals was shown to improve immune function and reduce the duration of upper respiratory infections in elderly subjects in nursing homes [141,142]. The immunogenic benefits were indicated by enhanced responses to the influenza vaccine (activation of specific T-lymphocyte subsets), reduced fever, and a reduction in newly prescribed antibiotics [141]. In contrast, feeding a prebiotic supplement containing FOS (70% Raftilose^®^ and 30% Raftiline^®^) to free-living seniors was confirmed to have no clear benefit on immune responses to vaccination [143]. A specific combination of long-chain inulin and oligofructose (Synergy1^®^) was also shown to impart limited effects on antibody responses to influenza vaccination in middle-aged subjects [144]. The variations in the formulation of prebiotic supplements in these studies could account for the discrepancies in the status of participants and outcomes. The type of prebiotic fiber, its complexity (purified or whole-plant) and added bio-actives tend to considerably influence the benefits achieved [77,78].

The established capacity of DF to influence gut microbiota and influence immune modulation indicates a promising protective potential against viral infections. The ability of DF to influence microbiota composition and undergo microbial fermentation to produce SCFAs, such as butyrate, acetate, and propionate, is frequently cited as the mechanism of action [87]. Mice fed a high-fiber diet rich in inulin had prolonged survival, reduced influenza virus-induced immunopathology, and improved anti-viral T-cell responses [55]. These dual effects strongly correlated to butyrate levels. A high-fiber diet was also confirmed to alter bone marrow hematopoiesis, leading to the accumulation of alternatively activated macrophages in the lungs of influenza virus-infected mice. The macrophages generated less CXCL1 chemokine, reducing early neutrophil influx into the airways and, thus, avoiding exaggerated tissue damage. The fermentation of DF also enhanced CD8+ T-cell metabolism, shown by increased activation, migration, cytotoxic activity, and viral clearance in high fiber-fed infected mice [55,145]. A protective role of SCFAs in modulating neutrophil migration in acute inflammation in the colon was also demonstrated in another study using acetate [146]. SCFAs modulated neutrophil migration, with acetate failing to suppress the accumulation of neutrophils in the intestinal tissues of GPR43−/− mice. The benefits of microbially-fermented SCFAs are suggested to be mediated through the direct actions of G-protein-coupled receptors (GPRs) expressed on the gut epithelium; adipose tissues; and immune cells, including monocytes and neutrophils [147]. These studies highlight the efficacy of DF supplementation in balancing the discrete innate, adaptive, and humoral immune components via SCFAs and setting an optimal immune tone in the airways to facilitate efficient viral clearance while avoiding exaggerated tissue damage [55].

The output of SCFAs varies widely depending on the type of DF as well as the presence of fiber-fermenting bacteria in the gut. In a study comparing the effect of three fermentable fibers on microbiota modulation and SCFA levels in humans, resistant potato starch was noted to be highly butyrogenic compared to inulin and resistant starch from maize [148]. Different bacterial changes were induced with potato resistant starch favoring the abundance of *Bifidobacteria* populations and butyrate-producing species. However, commonly studied purified DFs represent limited biochemical complexity compared to that occurring in fruits and vegetables [149]. Whole-plant prebiotic sugarcane fiber and green banana resistant starch flour supplements with natural fiber complexity have been shown to elevate SCFA levels and reduce local inflammation in acute colitis mice [77,78]. Non-purified DF supplements contain both soluble and insoluble fibers with rapid and slow-fermentable fractions and at ratios that more accurately represent those occurring in natural whole-plant foods [77]. DF supplementation could therefore be implemented in public health strategies for preventing and protecting against acute infections in addition to vaccination.

## 7. Synbiotics

The combination of PBs and prebiotic DFs, known as synbiotics, is receiving increased attention for its ability to confer augmented health benefits. A synbiotic is expected to impart augmented benefits to the host, owing to either complementary and/or synergistic functions [150]. The success of achieving the desired health effects with either PB or prebiotic DFs is dependent on several factors. PB benefits are highly species- and strain-specific. Moreover, regardless of the species/strain and the potential effects, the administered PB must survive gastric and bile acids and reach the intestine to impart the benefits associated with live organisms [75,151]. For sustained positive effects, PBs also need to be present from either continued ingestion or from having an effective prolonged residence time and replication in the gut. Furthermore, the ability of DF to induce its benefits via SCFA production is strictly dependent on the presence of fiber-fermenting gut microbiota [152]. Thus, the induction of substantial modulations in the complex network of microbiota and its metabolic capacity by specific PBs or prebiotics would be improved with a synbiotic. This involves simultaneous supplementation of the PB and prebiotic DF in combination to improve efficacy and health outcomes [77,78].

There is clinical evidence for synbiotic efficacy against VRIs that has been demonstrated in elderly populations, but the outcomes of this limited number of studies vary. A synbiotic with prebiotic galacto-oligosaccharide (GOS) and a bifidogenic growth factor in combination with heat-treated lactic acid-bacteria-fermented milk in elderly subjects (with history of stroke) enhanced vaccination response against influenza [153]. Synbiotic intervention in that study influenced *Bifidobacterium* counts and facilitated the sustainment of seroprotective effects for a longer period to the vaccine. In contrast, in another study, *B. longum bv. Infantis* CCUG 52486, combined with a prebiotic GOS, failed to reduce age-related changes in the NK cell response to seasonal influenza vaccination in elderly compared to young individuals [154]. There were only insignificant effects on B- and T-cell profiles in older subjects compared to those of younger counterparts [155]. The synbiotic combination increased memory IgA+, memory IgG+ and total IgG+ B cell levels in young subjects but failed to reproduce these effects in older participants and also ineffective in significantly altering T-cell subsets. These observations highlight the critical nature of immunosenescence, characterized by significant alteration in immune functions and gut dysbiosis in influencing the outcomes of interventions [156]. This might suggest that a more precise approach based on further research is required when selecting ingredients for synbiotic application in ageing. The application of senolytics, compounds that induce apoptosis in senescent cells, are currently undergoing clinical trials in humans for treating a variety of age-related pathologies [157,158]. Many senolytics, such as quercetin, fisetin, piperlongumine, and curcumin, have been identified as natural phytochemical compounds in foods [159]. Research efforts to determine the efficacy of combining senolytics and synbiotics in improving the age-related decline in immune functions could be explored.

**Figure 2:**
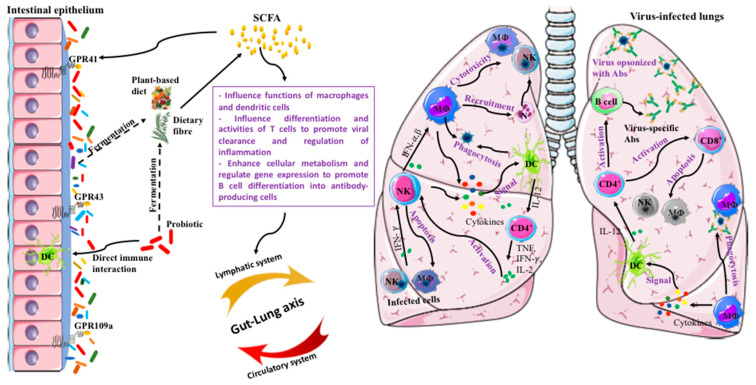
Potential mechanism by which synergistic synbiotic-induced short-chain fatty acids (SCFAs) can regulate the innate and acquired immune responses to attenuate viral respiratory infection.

Developing ARDS is responsible for high mortality among the elderly and individuals with chronic conditions and COVID-19 pneumonia [46]. Therefore, the designing of prophylactic nutritional strategies that facilitate mounting appropriate immune responses in at-risk individuals is prudent. The augmentation of SCFA production, for instance, could be a critical mechanism to support the development of more effective immunity (Figure 2). Gut-derived SCFAs have been shown to influence the functions of innate immune cells as well as impact acquired immune components. Notably, SCFAs are not restricted to the intestinal tract and are also disseminated through the circulatory system [160]. SCFAs produced by the bacteria are absorbed in the colon and either used by colonocytes or transported via the portal vein to reach the blood circulation and other organs. High fiber-induced SCFA production alters bone marrow hematopoiesis, characterized by the enhanced generation of macrophages and DC precursors and the subsequent seeding of the lungs by highly phagocytic DCs [145]. This type of DC was reported to have an impaired ability to induce Th2 cell effector function, thus resolving allergic airway inflammation in fiber-fed mice. In another study, the SCFA butyrate inhibited histone deacetylase 3 (HDAC3) to confer macrophages with non-inflammatory enhanced antimicrobial activity [161]. A shift in the macrophage metabolism, shown by the reduced glycolysis and inhibition of the mechanistic target of rapamycin (mTOR) activity, accounted for the butyrate-induced antimicrobial activity of the macrophages. Similarly, the ability of sodium butyrate to alleviate acute lung injury in mice has been shown through suppression of high-mobility group box 1 (HMGB1) release and NF-κB activation [162]. While the NF-κB activation promotes the heightened expression of inflammatory mediators in response to injury and inflammation, HMGB1 participates in the downstream development of acute lung injury as a late pro-inflammatory mediator. In addition, significant experimental evidence also supports the role of SCFAs in regulating the activities and differentiation of T-cells in impacting tissue inflammation [160]. Furthermore, SCFAs also accelerate cellular metabolism and regulate gene expression to promote plasma B cell differentiation in antibody-producing cells [54]. Hence, boosting SCFAs via microbial fermentation has promising potential in improving appropriate mucosal and systemic innate and acquired immune responses to control inflammation during infections and reinforce homeostasis.

Selecting a symbiotic combination using PBs that can potentiate elevated SCFAs by efficiently metabolizing the co-administered prebiotic DF, supporting other gut microbial fermenters, or prolonging the residence time of the PB in the gut should be considered. The declines in populations of primary and secondary fermenters in the inflamed gut is one of the prominent features of dysbiosis. In such conditions, the supplementation of only DF is insufficient to produce significant levels of SCFAs to impart benefits [152]. Similarly, probiotic intervention alone may be of limited efficacy in the absence of fiber substrate to influence the growth and activity of the administered probiotic. Probiotic spores, in synbiotic combination with whole-plant prebiotic DF, augmented butyrate production along the entire colon length [77,78]. In contrast, neither PB *B. coagulans* or prebiotic alone could produce the same level of this effect in a chemically induced mice model of colitis. The ability of *B. coagulans* to metabolize a variety of plant fibers to produce SCFAs and lactic acid has been previously determined [163,164,165]. *B. coagulans* spores in synbiotic combination with whole-plant sugar cane fibre [77] or green banana resistant starch [78] induced protection against chemically induced damage to the colonic epithelium by reducing inflammatory infiltrates and preventing the disruption of tight junction proteins, along with augmentation of SCFA levels. The administration of *B. coagulans*, in combination with FOS and GOS in the elderly, has also been shown to result in beneficial microbial changes in fecal microbiota ex vivo [166], while another in vivo study [167] identified a suitable synbiotic combination to enhance the butyrate production in swine, inducing improved immune functions. Potato resistant starch was reported to stimulate lactic acid bacteria and secondary fermenters, such as *Anaerostipes hadrus*, in the GI tract of swine, increasing the SCFA production. Thus, a synbiotic that can deliver a fermentable DF and a PB that can metabolize it is an attractive strategy to support optimal immune responses against the immunopathology of VRIs (Figure 2).

## 8. Polyphenolic Plant Bioactives

In addition to DF, phytochemicals including vitamins, micronutrients, and polyphenols present in fruits and vegetables are ingested as a part of the diet. Phytochemicals have similarly been shown to be of considerable importance in nutritional strategies for addressing the severity of viral respiratory diseases [62,168,169]. Apart from the ability of certain polyphenols to influence microbiota composition, they also impart antioxidant, anti-inflammatory, and anti-viral effects [170,171,172]. The antiviral effects of polyphenols have been demonstrated to be mediated either by direct inhibitory effects on virus replication or through the induction of immunomodulatory/antioxidant responses [170,171]. Oxidative stress has been implicated in lung tissue injury and epithelial barrier dysfunction in acute respiratory viral infections [31,32]. Accumulating evidence has revealed the excellent immunomodulatory effects of epigallocatechin-3-gallate (EGCG), abundant in green tea on both innate and adaptive immune responses [173]. EGCG supplementation has been proven experimentally to alleviate acute lung injury induced by swine influenza virus via the inhibition of TLR4 signaling and reducing inflammatory cell migration [174]. Green tea catechin metabolites have been shown to enhance CD4+ T-cell and NK cell activities in vivo [175]. Moreover, polyphenols upon reaching the colon are also known to interact with gut microbiota [176], thus influencing their metabolic and immune capabilities [177]. While this evidence from experimental studies indicates the substantial capacity of plant-based bioactives in influencing immune functions and microbiota, the limited studies in viral-related human disease highlights the need for more research in this area.

## 9. Conclusions

There is strong evidence to support the integral role of the microbiota in shaping host immunity. The severe immunopathology of VRIs, including tissue damage, pneumonia, ARDS, multiorgan failure, and death, is induced by dysregulated immune responses and exacerbated inflammation. Moreover, underlying chronic inflammation and associated dysbiosis increases the risk of morbidity and mortality. Employing combined PB, DF, and nutritionally sourced plant bioactives to modulate bacterial composition and activities is a pragmatic approach for enhanced protection against the acute morbidities associated with VRIs. The use of selected synergistic combinations of compatible PB, DF, and bioactives might be capable of improving consistency in balancing innate and acquired immune responses at appropriate levels for viral clearance and to reinforce homeostasis. The evidence from experimental studies on their ability to influence immune health and disease severity in VRIs warrants more defined clinical studies to confirm the benefits in humans. This knowledge will potentially guide public health recommendations and the development of dietary strategies targeted at enhanced immune fitness and wellbeing in response to acute viral challenges.
